# Ribonuclease 5 facilitates corneal endothelial wound healing via activation of PI3-kinase/Akt pathway

**DOI:** 10.1038/srep31162

**Published:** 2016-08-16

**Authors:** Kyoung Woo Kim, Soo Hyun Park, Soo Jin Lee, Jae Chan Kim

**Affiliations:** 1 Department of Ophthalmology, College of Medicine, Chung-Ang University Hospital, Seoul, Korea; 2 Graduate School of Chung-Ang University, College of Medicine, Seoul, Korea

## Abstract

To maintain corneal transparency, corneal endothelial cells (CECs) exert a pump function against aqueous inflow. However, human CECs are arrested in the G_1_-phase and non-proliferative *in vivo.* Thus, treatment of corneal endothelial decompensation is limited to corneal transplantation, and grafts are vulnerable to immune rejection. Here, we show that ribonuclease (RNase) 5 is more highly expressed in normal human CECs compared to decompensated tissues. Furthermore, RNase 5 up-regulated survival of CECs and accelerated corneal endothelial wound healing in an *in vitro* wound of human CECs and an *in vivo* cryo-damaged rabbit model. RNase 5 treatment rapidly induced accumulation of cytoplasmic RNase 5 into the nucleus, and activated PI3-kinase/Akt pathway in human CECs. Moreover, inhibition of nuclear translocation of RNase 5 using neomycin reversed RNase 5-induced Akt activation. As a potential strategy for proliferation enhancement, RNase 5 increased the population of 5-bromo-2′-deoxyuridine (BrdU)-incorporated proliferating CECs with concomitant PI3-kinase/Akt activation, especially in CECs deprived of contact-inhibition. Specifically, RNase 5 suppressed p27 and up-regulated cyclin D1, D3, and E by activating PI3-kinase/Akt in CECs to initiate cell cycle progression. Together, our data indicate that RNase 5 facilitates corneal endothelial wound healing, and identify RNase 5 as a novel target for therapeutic exploitation.

In mammals, the transparent and avascular cornea serves as an optical window that allows entry of light and images for optimal vision. To maintain corneal transparency against the inflow of aqueous humor into the corneal stroma, the cornea utilizes a homeostatic system. Among the three different types of cells comprising the cornea, namely, epithelial, stromal and endothelial cells, corneal endothelial cells (CECs) form the innermost monolayer of the cornea in a well-arranged mosaic pattern that acts as a water barrier due to the presence of ionic pumps^1^.

Human CECs are arrested in the G_1_-phase of the cell cycle^2^ due to several negative regulators suppressing the S-phase entry^3^,^4^, and thus do not proliferate *in vivo*. Instead, *in vivo* CECs overcome minor corneal endothelial damage caused by ocular trauma, intraocular surgery, diabetes, or glaucoma via the migration of adjacent CECs and cell enlargement rather than mitosis^5^,^6^. Unfortunately, if the density of human CECs drops below a critical threshold (400–500 cell/mm^2^) as a result of serious injury or endothelial dystrophy, the decompensated barrier function of the corneal endothelium results in corneal edema with tearing, recurring pain, and eventual vision loss. Corneal transplantation or keratoplasty is currently the only therapeutic option with a favorable outcome^7^. Nevertheless, the density of CECs decreases rapidly in the first 6 months postoperative before slowing to a rate that still exceeds the physiological loss rate of 0.6% per year^8^. Moreover, corneal transplantation may cause severe complications, such as graft failure and allograft rejection, which may require re-grafting^9^,^10^. Lastly, there continues to be a worldwide shortage of donor corneas.

A number of studies have reported evidence supporting the proliferative capacity of human CECs in *ex vivo* corneas^11^,^12^. In addition, a subpopulation of CECs with potential proliferative potential at the corneal endothelial periphery was recently reported^13^,^14^, suggesting that triggering and controlling the proliferation of CECs could serve as a therapeutic bioengineering approach for treating corneal endothelial dysfunction.

Ribonuclease (RNase) 5, commonly known as angiogenin (ANG), is a 14.4-kDa single-chain protein originally identified as a potent inducer of neovascularization^15^. ANG translocates to the nucleus, where it binds ribosomal DNA and stimulates the transcription of rRNA^16^,^17^,^18^,^19^ for diverse functions beyond angiogenesis. Thus, ANG protein is now referred to as RNase 5, and is the 5^th^ member of RNase A family^20^. Although ANG is up-regulated in a variety of human cancers^21^,^22^,^23^,^24^,^25^,^26^,^27^,^28^, there have been recent reports of a neuroprotective role of RNase 5 in neurodegenerative disorders^29^,^30^,^31^,^32^,^33^,^34^,^35^ and a novel action of RNase 5 for promoting cell survival under stress conditions^20^,^36^, suggesting that RNase 5 may be an essential homeostatic factor. In ophthalmology research, in a similar vein, RNase 5 appears to be normally present in human tear fluid and may participate in the host defense system^37^.

We hypothesized that human CECs may be physiologically armed with self-protective mechanisms against aging and injury, including surgery, trauma, and the lifelong shear stress of aqueous flow in corneal endothelium generated by rapid eye movements during sleep^38^ and convection flow in the anterior chamber. To explore this hypothesis, we investigated the expression of RNase 5, a candidate homeostatic factor, in normal and decompensated human corneal endothelial tissue. We then investigated potential molecular signals of RNase 5 involved in cellular proliferation, consisting of phosphatidylinositol 3-kinase (PI3-k) activation^39^ and nuclear translocation of RNase 5^16^.

In the present study, we identified the molecular mechanisms required for G_1_/S progression which is subsequently provoked by RNase 5 for *in vitro* proliferation of human CECs. We also investigated the role of RNase 5 in corneal endothelial wound healing in a rabbit model of transcorneal cryogenic endothelial injury. Together, our findings suggest that RNase 5, by virtue of its ability to induce CECs to exit the cell cycle, may be a novel treatment approach for corneal endothelial dysfunction.

## Results

### RNase 5 is distributed to the cytoplasm of CECs in *ex vivo* normal human corneal endothelium and, by contrast, is deficient in decompensated corneal endothelial tissues

**Figure 1:**
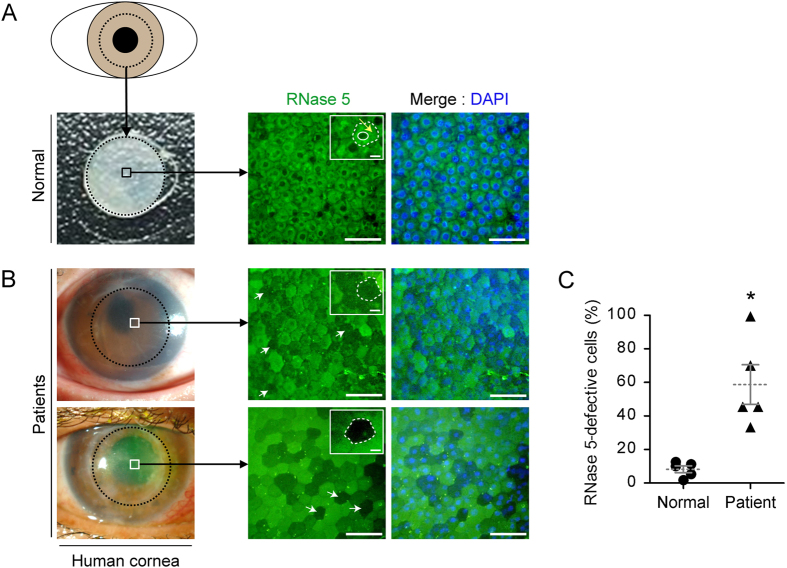
*Ex vivo* expression patterns of ribonuclease (RNase) 5 protein in normal and decompensated human corneal endothelial tissues. (**A,B**) Representative images showing flat-mounted immunofluorescence (IF) staining of RNase 5 protein in normal (**A**) and decompensated human corneal endothelial tissues (**B**). Central circular corneal tissues with a diameter of approximately 8 mm (dotted black circles) were punched and excised from donor corneas. Thereafter, RNase 5 protein-expressing corneal endothelial cells (CECs) in the central area of corneas (squares in left column) were microscopically evaluated by IF. Protein expression of RNase 5 was clear in CECs of normal tissue, but defective in CECs of decompensated tissues (white arrows, shown representatively). In the magnified views (small white rectangles in middle column), normal CECs exhibited copious expression of RNase 5 protein in the cytoplasm (yellow arrow, **A**) between the CEC contour (dotted white circle) and nucleus (white solid circle). On the other hand, the expression of RNase 5 protein was defective by IF stain in CECs in decompensated human corneal endothelial tissues (dotted white circles, **B**). (**B**) Photos in the left column are snapshots of a cornea taken prior to surgical excision for corneal transplantation. Scale bar: 50 μm except for those in small white rectangles, which are 10 μm. (**C**) The proportion of CECs that exhibited defective expression of RNase 5 protein according to IF analysis was significantly higher in corneal tissues from patients with decompensated corneal endothelium. *p = 0.012, vs. normal (*t*-test). *n* = 5 corneas in each group. Values represent the mean ± s.e.m.

RNase 5 is a candidate survival factor for CECs based on their embryonic origin from the neural crest^40^ and its recently discovered neuroprotective role. We compared the expression of RNase 5 in human corneal endothelial tissues from deceased subjects without previous cancer or ocular disorders to RNase 5 expression in excised corneal endothelial tissues from patients with corneal endothelial decompensation caused by pseudophakic bullous keratopathy, infection, or burns obtained during keratoplasty. In normal corneal endothelial tissues, most CECs revealed moderate immunofluorescence of RNase 5 (Fig. 1A). Moreover, the intracellular expression of RNase 5 protein was relatively focused in the cytoplasm. On the contrary, decompensated corneal endothelial tissues contained CECs with decreased immunofluorescence of intracellular RNase 5 protein (Fig. 1B). The overall proportion of RNase 5 protein-defective CECs was significantly higher (*p* = 0.012) in diseased corneal endothelial tissues (Fig. 1C).

### RNase 5 enhances survival of human CECs

**Figure 2:**
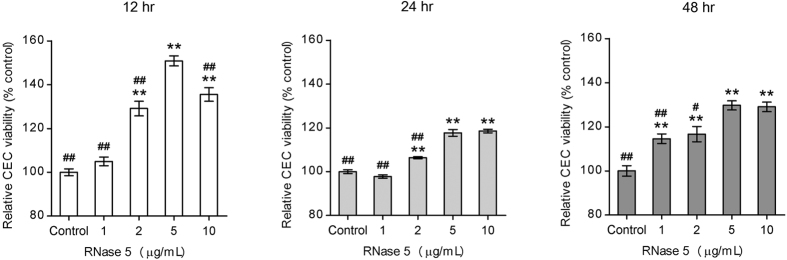
Dose-dependent cell viability (MTT assay) of human corneal endothelial cells (CECs) treated with ribonuclease (RNase) 5. Relative CEC viability compared to controls over 48 hours according to the concentration of RNase 5. The number of viable CECs was significantly elevated compared to control by RNase 5, and the lowest concentration of RNase 5 showing maximal CEC viability was 5 μg/mL at all three timepoints (12-, 24- and 48-hour treatments). **p < 0.01, vs. control; ^##^*p* < 0.01 and ^#^*p* < 0.05, vs. 5 μg/mL of RNase 5 (ANOVA followed by Bonferroni’s *post-hoc* analysis). *n* = 4 independent experiments at all three timepoints. Values represent the mean ± s.e.m.

RNase 5 has been proposed to mediate both cell survival and growth mechanisms in neurodegenerative disease^41^. To verify that RNase 5 enhances survival in human CECs, cell viability was measured using the MTT assay in the presence or absence of RNase 5. The number of viable cells was monitored over 48 hours after the start of the culture. Viability of CECs increased over a wide range of RNase 5 concentrations, from 1 to 10 μg/mL, and after culturing for 12, 24 and 48 hours. The effect of cell-survival enhancement by RNase 5 compared to control was noted at all three timepoints (Fig. 2). A concentration of 5 μg/mL, which showed most prominent effect on enhancing CEC viability at all timepoints, was selected for subsequent use in this study.

### RNase 5 promotes wound healing in an *in vitro* model of human CECs and an *in vivo* rabbit model

**Figure 3:**
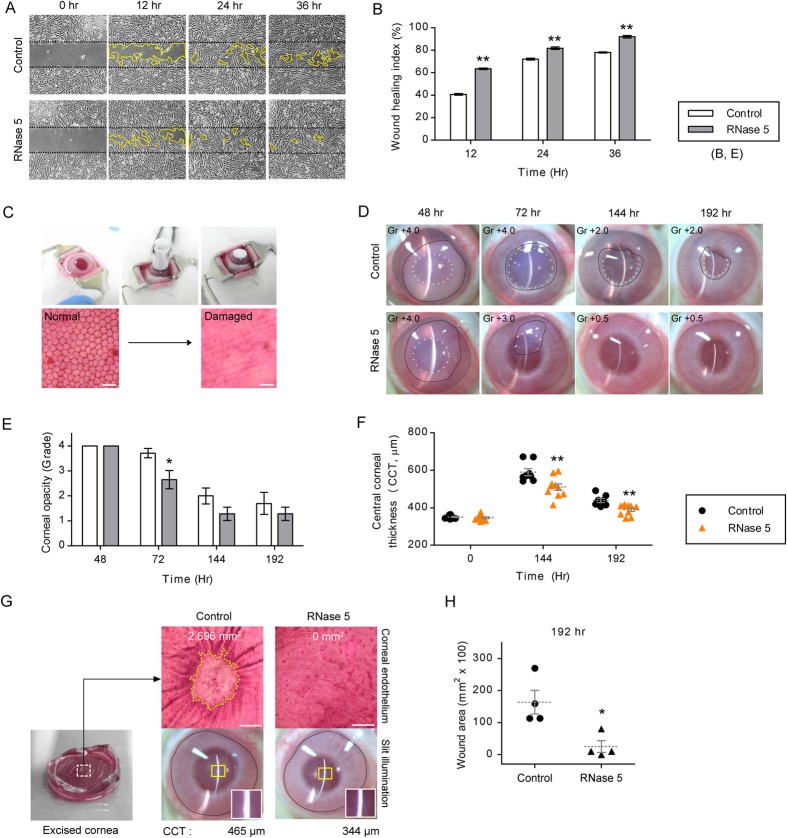
Enhancement of corneal endothelial wound healing by ribonuclease (RNase) 5 treatment using *in vitro* human corneal endothelial cells (CEC) and an *in vivo* rabbit wound model. (**A,B**) *In vitro* wound healing of cultured CECs with or without RNase 5 (5 μg/mL). (**A**) The remaining wound area (boundary of yellow line) is shown in both groups. (**B**) The wound healing index was significantly higher in the RNase 5 group. **p = 2.51e-5 (12 hr); **p = 0.002 (24 hr); **p = 3.74e-4 (36 hr), vs. control (*t*-test). *n* = 3 independent experiments. (**C**) Establishment of a transcorneal cryo-freezing endothelial wound model in rabbits. Scale bar: 40 μm. (**D**) Representative photos showing the corneal opacity with or without RNase 5 eye drops in rabbits. The corneal opacity (boundary of black line) was more prominent in the control from 72 hours. The iris configuration at pupil margin was completely obscured (grade [Gr] + 4.0) in both groups at 48 hours. Thereafter, the sectorial width of iris contour obscured by corneal opacity (white dotted line) was less at 72 hours (Gr + 3.0) and disappeared from 144 hours (Gr + 0.5) in the RNase 5 group. (**E,F**) Quantification of corneal opacity and central corneal thickness (CCT) in the control and RNase 5 group. (**E**) *p = 0.022 (72 hr), vs. control (*t*-test). (**F**) **p = 0.003 (144 hr); **p = 0.001 (192 hr), vs. control (*t*-test). *n* = 7–10 eyes per group. (**G**) Representative photos showing the rabbit corneal endothelial wound area and CCT. The remnant wound area by Alizarin red S stain (boundary of yellow dotted line) was 2.696 mm^2^ at 192 hours in the control, whereas completely healed in the RNase 5 group. The central slit beam (white squares magnified from yellow rectangles) was thicker and the relevant CCT quantified by pachymetry was higher in the control (465 μm). Scale bar: 1 mm. (**H**) Comparison of corneal endothelial wound area (192 hr) between the control and RNase 5 group. *p = 0.015 (192 hr), vs. control (*t*-test). *n* = 4 eyes per group. Values represent the mean ± s.e.m.

To evaluate the potential effect of RNase 5 on corneal endothelial wound restoration, we administered RNase 5 to an *in vitro* scrape wound model of cultured human CECs and applied RNase 5 eye drops to an *in vivo* rabbit corneal endothelial wound model. A time-course treatment with RNase 5 (5 μg/mL) enhanced the migration capacity (wound healing index) of CECs significantly compared with the untreated control (12 hr, *p* = 2.51e-5; 24 hr, *p* = 0.002; 36 hr, *p* = 3.74e-4) (Fig. 3A,B). Although wound healing of CECs pretreated with mitomycin C also tended to be higher in the RNase 5 group, the wound healing index did not differ significantly between both groups (Supplementary Fig. S1), which suggests that RNase 5-induced enhancement of *in vitro* wound healing may be attributed to the proliferation more than the migration.

To test whether RNase 5 actually enhanced *in vivo* corneal endothelial wound healing, we used a rabbit model of transcorneal freezing injury of the corneal endothelium, in which the corneal endothelial layer was destroyed by freezing (Fig. 3C). Treatment with RNase 5 eye drops significantly reduced the corneal haziness (Fig. 3D,E) compared to controls at 72 hours after injury (*p* = 0.022), and the inter-group difference was maintained thereafter, but insignificant. Moreover, RNase 5 significantly lowered central corneal thickness (CCT), which indicates decrease of corneal edema at 144 and 192 hours (*p* = 0.003 and 0.001, respectively) after injury (Fig. 3F).

To investigate whether RNase 5 could promote corneal endothelial wound healing in an *in vivo* rabbit model, the wound area in endothelial layer was evaluated by Alizarin red S staining after enucleation at a maximal follow-up period (192 hours). The mean wound area of the RNase 5 group was significantly smaller than that of the control group (*p* = 0.015) (Fig. 3G–H).

### RNase 5 treatment induces nuclear accumulation of RNase 5 in human corneal endothelial tissues and in CECs

**Figure 4:**
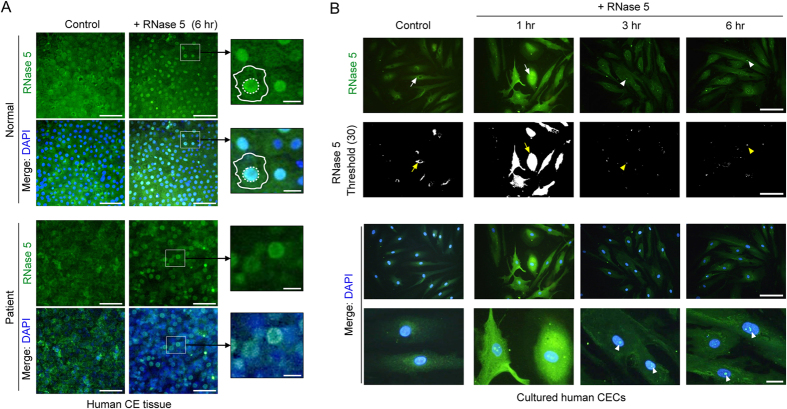
Ribonuclease (RNase) 5 treatment-induced nuclear localization of RNase 5 in human corneal endothelium tissues and cultured human corneal endothelial cells (CECs). (**A**) Representative images showing flat-mounted immunofluorescence stain of RNase 5 in normal and decompensated human corneal endothelial tissue with or without RNase 5 treatment. RNase 5 was predominantly expressed in the nucleus after 6-hour treatment with exogenous human RNase 5 (5 μg/mL) in normal and decompensated corneal endothelium (right column). In magnified images of small white rectangles, the contour of CECs (white border lines) and the nuclear expression of RNase 5 after RNase 5 treatment (white dotted circles) are outlined. Scale bar: 50 μm (white) and 10 μm (yellow). *n* = 3 independent experiments. (**B**) RNase 5 localization in cultured human CECs with or without RNase 5 treatment. RNase 5 expression was detected at the cytoplasmic perinuclear area in untreated CECs (arrows in the control column). After RNase 5 treatment, intracellular RNase 5 expression became very prominent early (arrows in 1 hr column), and thereafter localized specifically to the nuclei (arrow heads in 3 hr and 6 hr columns). This observation was more apparent with thresholding (upper second row, lightness 30). Scale bar: 100 μm (three upper rows), 20 μm (lowest row). *n* = 3 independent experiments.

It has been reported that RNase 5 exhibits differential subcellular localizations in HeLa cells, being localized to either the nucleus with nucleolar accumulation or the cytoplasm under growth and stress conditions, respectively^42^. During growth, RNase 5 accumulates in the nucleus where it stimulates rRNA transcription in the vasculature system^18^,^43^. To verify this phenomenon in CECs, which express RNase 5 as shown in Fig. 1, we evaluated the immunofluorescence of RNase 5 in *ex vivo* human corneal endothelial tissues after treatment with exogenous human RNase 5. In both normal and decompensated human corneal endothelial tissues, exogenous RNase 5 treatment (5 μg/mL for 6 hours) induced RNase 5 accumulation in the nucleus (Fig. 4A).

To elucidate the mechanism of this phenomenon, we confirmed that RNase 5-induced nuclear accumulation of RNase 5 in cultured human CECs. Prior to beginning the experiments, the corneal endothelial phenotype of cultured human CECs was identified by the presence and localization of Na^+^/K^+^-ATPase and zonula occludens (ZO)-1, which are normally expressed along cell membrane specifically in CECs among cells comprising a cornea, thus are famous markers of CECs (Supplementary Fig. S2).

RNase 5 was detected predominantly in the cytoplasmic perinuclear area in untreated CECs (Fig. 4B, arrow in column control). Treatment with RNase 5 (5 μg/mL) induced copious cytoplasmic staining of RNase 5 (Fig. 4B, arrow in column 1 hr) with intranuclear localization after incubation for 1 hour. Thereafter, the nuclear expression of RNase 5 maintained (Fig. 4B, arrow heads in column 3 and 6 hr) with attenuated staining in the cytoplasm after incubation for 3 and 6 hours. These results indicated that CECs may utilize RNase 5-elicited intracellular signaling. Furthermore, these data suggested that the localization of RNase 5 by exogenous application of RNase 5 varies in a time-dependent manner, with localization to the cytoplasm within 1 hour and to the nucleus of CECs thereafter.

### RNase 5 activates Akt in CECs and nuclear translocation of RNase 5 is involved in RNase 5-induced Akt activation

**Figure 5:**
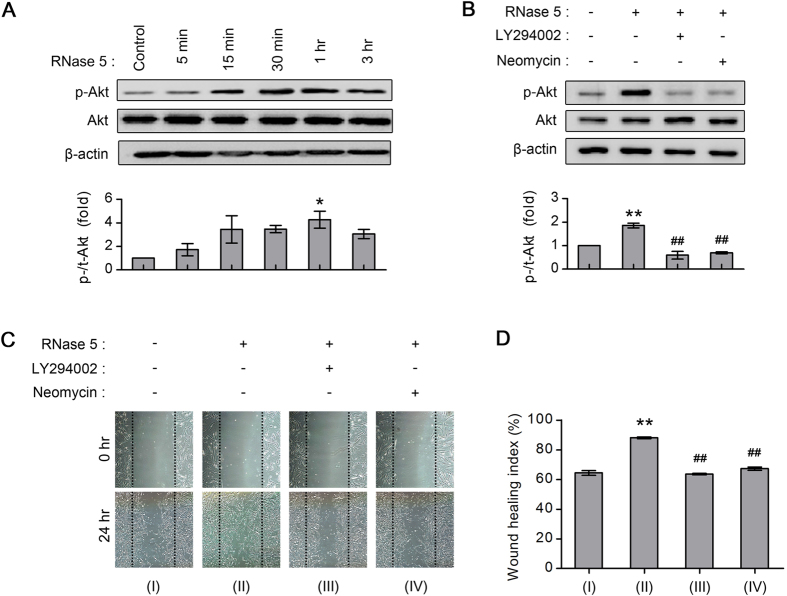
Ribonuclease (RNase) 5 activates PI3-kinase (PI3-k)/Akt signal pathway in human corneal endothelial cells (CECs) for wound healing enhancement and the nuclear translocation of RNase 5 is associated with RNase 5-induced PI3-k/Akt activation. (**A**) Representative immunoblotting of phosphorylated (p-Akt) and total Akt and quantitative analysis of the band density of relative p-Akt in cultured human CECs before (control) and after RNase 5 treatment (5 μg/mL, for 5 minutes to 3 hours). *p = 0.037, vs. control. *n* = 4 independent experiments. (**B**) Representative immunoblotting of p-Akt and total Akt and quantitative analysis of the band density of relative p-Akt in cultured human CECs with or without RNase 5 (5 μg/mL, 24 hours), and in the presence or absence of LY294002 (20 μM, 1 hour) or neomycin (1 mM) co-treatment. **p = 0.002, vs. control. ^##^*p* = 1.11e-4, RNase 5 treatment with vs. without LY294002. ^##^*p* = 2.06e-4, RNase 5 treatment with vs. without neomycin. *n* = 3 independent experiments. (**A,B**) β-actin was used as the loading control for Western blots. Full-length gels are presented in Supplementary Fig. S5. (**C,D**) The *in vitro* wound healing in a scraped wound model of cultured human CECs with or without RNase 5 (5 μg/mL, 24 hours), and in the presence or absence of LY294002 (20 μM) or neomycin (1 mM) co-treatment. (**D**) LY294002 or neomycin significantly inhibited RNase 5-induced wound healing enhancement. **p = 1.33e-6, vs. control. ^##^*p* = 1.03e-6, RNase 5 treatment with vs. without LY294002. ^##^*p* = 3.61e-6, RNase 5 treatment with vs. without neomycin. *n* = 3 independent experiments. (**A,B,D**) Statistical analysis was performed with ANOVA followed by Bonferroni’s *post-hoc* analysis. Values represent the mean ± s.e.m.

The PI3-k/Akt pathway is centrally involved in cell proliferation^44^. In a previous study, RNase 5 was shown to promote human umbilical vein cell migration and induce angiogenesis in chick chorioallantotic membranes via phosphorylation of Akt, an important downstream effector of PI3-k^45^. Although a few reports have stressed the involvement of the PI3-k/Akt pathway for human CEC proliferation^46^,^47^, there have been no studies regarding RNase 5-induced Akt activation in CECs. In this study, RNase 5 (5 μg/mL) increased Akt phosphorylation in cultured human CECs in a time-dependent manner (Fig. 5A). Notably, Akt, a representative subcellular cytoplasmic signaling molecule, is activated within one hour of RNase 5 treatment, consistent with the timing of intracytoplasmic RNase 5 localization as shown in Fig. 4B.

It was reported that neomycin inhibited nuclear translocation of RNase 5 in human umbilical vein endothelial cells and RNase 5-induced cell proliferation for angiogenesis^48^. Likely, the treatment of CECs with neomycin suppressed RNase 5-induced nuclear accumulation of RNase 5 (Supplementary Fig. S3), and reversed RNase 5-induced increase of ratio of nuclear amount of RNase 5 to cytoplasmic amount (N/C expression ratio) (Supplementary Fig. S4). We hypothesized that intranuclear translocation of RNase 5 may be involved in the activation of the PI3-k/Akt pathway in CECs. Co-treatment with neomycin (1 mM) inhibited RNase 5-induced phosphorylation of Akt, like PI3-k inhibitor (LY294002, 20 μM) did (Fig. 5B), indicating that RNase 5-induced Akt activation is regulated by nuclear translocation of RNase 5 in CECs.

### PI3-k/Akt activation is associated with RNase 5-induced enhancement of *in vitro* wound healing of human CECs

*In vitro* CEC migration was evaluated with or without RNase 5 (5 μg/mL), and in the presence or absence of LY294002 (20 μM) or neomycin (1 mM) co-treatment to verify the effect of RNase 5-induced, and nuclear translocation of RNase 5-related Akt activation (as noted in Fig. 5A,B) on *in vitro* wound healing. LY294002 or neomycin co-treatment inhibited the effect of CEC migration enhancement induced by RNase 5 treatment to the level of control at 24 hours (Fig. 5C,D), suggesting that PI3-k/Akt activation may play a key role in the signaling of RNase 5-enhanced corneal endothelial wound healing.

### RNase 5 up-regulates mitosis-related cell cycle signaling in human CECs

**Figure 6:**
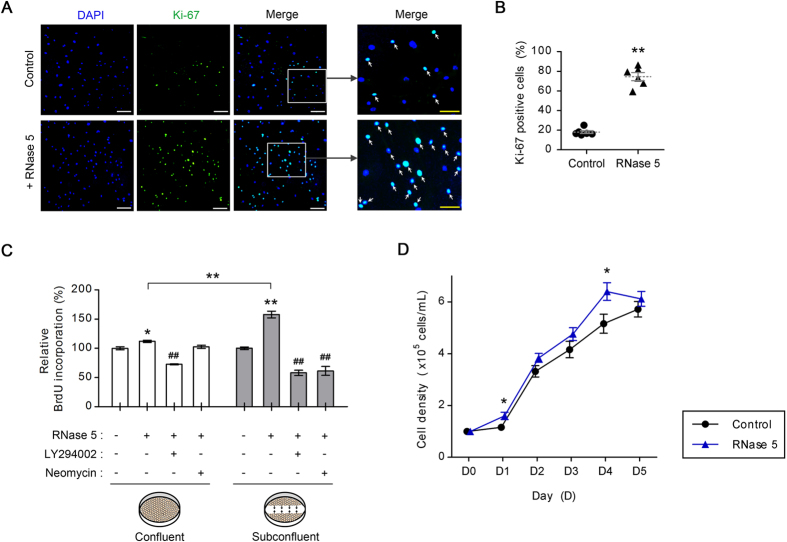
Effect of ribonuclease (RNase) 5 on the proliferation of cultured human corneal endothelial cells (CECs). (**A,B**) Representative images of Ki-67 staining of cultured human CECs with or without RNase 5 treatment (5 μg/mL, 24 hours, **A**) and comparison of proportion of Ki-67^+^ cells between each group (**B**). (**A**) In magnified images of small white rectangles, the Ki-67^+^ cells with or without RNase 5 treatment are indicated (white arrows). Scale bar: 200 μm (white) and 100 μm (yellow). *n* = 3 independent experiments. (**B**) **p = 8.52e-6, vs. control (*t*-test). *n* = 6 independent experiments. (**C**) Relative BrdU incorporation in cultured human CECs with RNase 5 treatment (5 μg/mL, 24 hours) and LY294002 (20 μM) or neomycin (1 mM) co-treatment compared to control. RNase 5 elevated BrdU incorporation in CECs more prominently in cells of the scraping wound model compared with confluent cells. Confluent (ANOVA followed by Bonferroni’s *post-hoc* analysis): *p = 0.023, vs. control; ^##^*p* = 6.04e-6, RNase 5 treatment with vs. without LY294002. Subconfluent (ANOVA followed by Bonferroni’s *post-hoc* analysis): **p = 3.43e-4, vs. control; ^##^*p* = 5.84e-6, RNase 5 treatment with vs. without LY294002; ^##^*p* = 7.60e-6, RNase 5 treatment with vs. without neomycin. **p = 0.001, RNase 5 treatment in confluent vs. scrape wound model (*t*-test). *n* = 3 independent experiments. (**D**) Growth curves of CECs with or without RNase 5 treatment (5 μg/mL). *p = 0.043, vs. control (D0); *p = 0.038, vs. control (D4) (*t*-test). *n* = 5 independent experiments. Values represent the mean ± s.e.m.

The most desirable strategy for corneal endothelial wound healing is to repair the corneal endothelium by releasing CECs from cell-cycle arrest and replicating residual CECs to replace dead or injured cells. In this context, we explored the possibility of RNase 5-elicited cell cycle signaling for proliferation of human CECs by analyzing Ki-67 expression and 5-bromo-2′-deoxyuridine (BrdU) incorporation into DNA. Subconfluent CECs were treated with RNase 5 (5 μg/mL for 24 hours) and Ki-67^+^ cells were counted and compared to control CECs to analyze differences in proliferation. The population of Ki-67^+^ cells after treatment with RNase 5 was significantly increased to approximately 75% among the total population, compared to 18% in the absence of RNase 5 (*p* = 8.52e-6) (Fig. 6A,B). Moreover, the proliferative effect of RNase 5 was confirmed using an *in vitro* scrape wound model. Interestingly, RNase 5 elevated Ki-67 expression, which was mainly confined to the initial margin of the wound where migrating CECs were concentrated and not subject to contact-inhibition (Supplementary Fig. S6).

Although it is still not fully understood what mediates the inhibition of CEC proliferation in mature corneal endothelia, contact-inhibition is a strong candidate^49^. Thus, we treated cultured CECs with RNase 5 under two different conditions: 1) fully confluent and 2) scrape-wound subconfluent plated CECs. Proliferation, assessed by BrdU incorporation, increased with RNase 5 (5 μg/mL for 24 hours) in confluent and subconfluent conditions by approximately 10% and 60%, respectively (Fig. 6C). Moreover, blockade of either PI3-k/Akt signaling by LY294002 or nuclear translocation of RNase 5 by neomycin, significantly inhibited RNase5-induced proliferation in subconfluent CECs under scrape wound condition (*p* = 5.84e-6 and 7.60e-6, respectively). The increase of BrdU incorporation by RNase 5 was significantly higher in subconfluent CECs (*p* = 0.001) compared to fully confluent cells. Based on these results, we hypothesized that RNase 5 may have a sufficient mitotic effect on CECs under wound conditions where there is a loss of contact-inhibition.

The velocity of CEC growth either in the absence or in the presence of RNase 5 was subsequently verified to evaluate the effect of RNase 5 on the eventual proliferation of CECs. According to daily growth curves of CECs, RNase 5 enhanced CEC growth compared to control with significance at initial growth (day 1) and at close to being confluent (day 4) (Fig. 6D).

### RNase 5 mediates p27Kip1 phosphorylation and upregulation of cyclin D1, D3, and E via activation of PI3-k/Akt pathway

**Figure 7:**
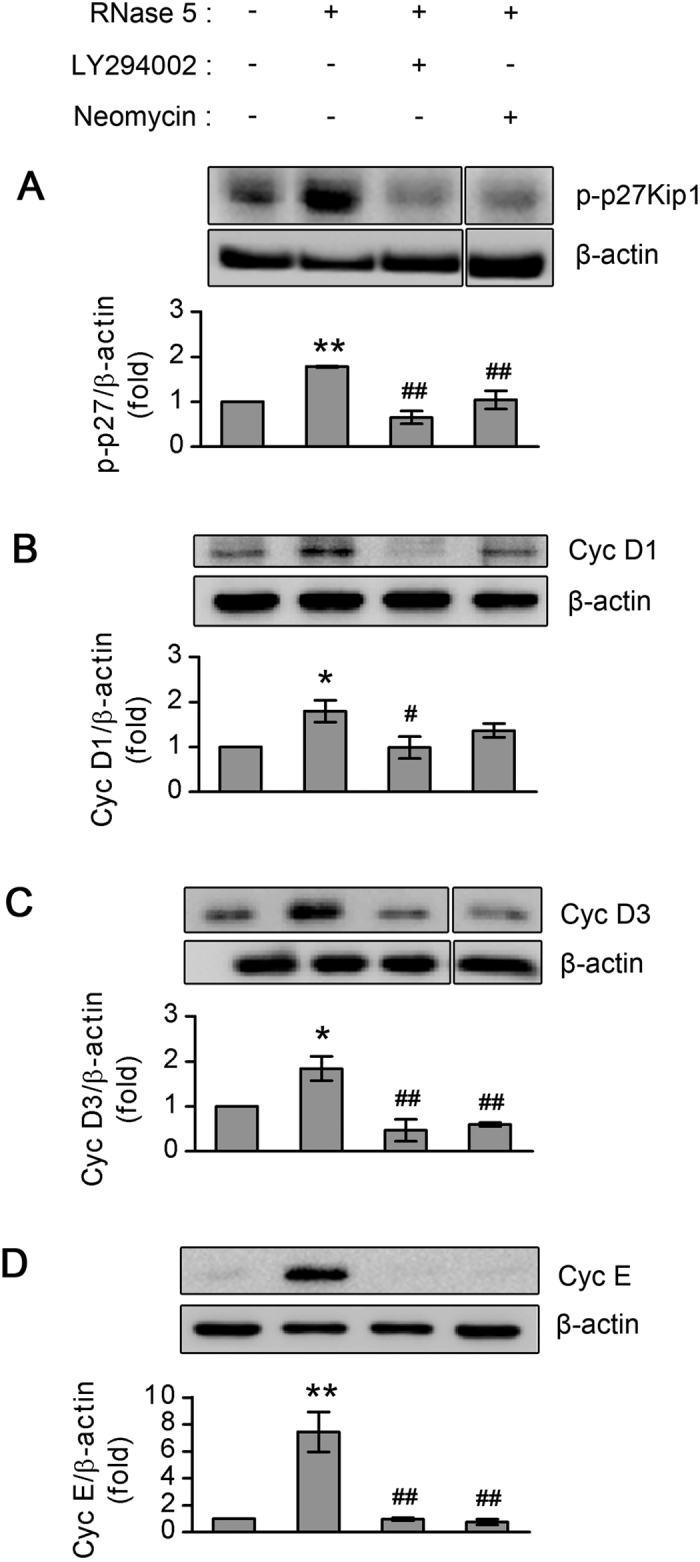
The effect of ribonuclease (RNase) 5 on G_1_ to S cell cycle progression via PI3-kinase/Akt activation in cultured human corneal endothelial cells (CECs). (**A–D**) Representative immunoblotting and quantitative analysis of band density of phosphorylated p27Kip1 (p-p27Kip1, **A**), cyclin (Cyc) D1 (**B**), cyc D3 (**C**) and cyc E (**D**) in cultured human CECs with or without RNase 5 (5 μg/mL, 24 hours), and in the presence or absence of LY294002 (20 μM) or neomycin (1 mM) co-treatment. P27Kip1 (**A**) **p = 0.005, vs. control; ^##^*p* = 1.86e-4, RNase 5 treatment with vs. without LY294002; ^##^*p* = 0.007, RNase 5 treatment with or without neomycin. Cyc D1 (**B**) *p = 0.046, vs. control; ^#^*p* = 0.042, RNase 5 treatment with vs. without LY294002. Cyc D3 (**C**) *p = 0.042, vs. control; ^##^*p* = 0.001, RNase 5 treatment with vs. without LY294002; ^##^*p* = 0.003, RNase 5 treatment with vs. without neomycin. Cyc E (**D**) **p = 3.63e-4, vs. control; ^##^*p* = 3.45e-4, RNase 5 treatment with vs. without LY294002; ^##^*p* = 2.53e-4, RNase 5 treatment with or without neomycin. Statistical analysis was performed with ANOVA followed by Bonferroni’s *post-hoc* analysis. *n* = 4 independent experiments. β-actin was used as a loading control for Western blotting, and the small gaps indicate skipped lanes from the same membrane. Values represent the mean ± s.e.m. Full-length gels are presented in Supplementary Fig. S7.

P27Kip1 protein binds to and prevents the activation of cyclin E-CDK2 or cyclin D-CDK4 complexes, thus inhibiting the S phase progression. Activation of cyclin D in early G_1_ and cyclin E in late G_1_ promote S phase entry and progression, respectively, as positive G_1_ regulators^11^. The abundance of p27 is regulated by its phosphorylation and subsequent ubiquitin-proteasome-mediated degradation^47^. Thus, phosphorylation of p27 is a prerequisite for degradation of p27 and a major mechanism for G_1_/S cell cycle progression^50^. Importantly, PI3-k/Akt signaling is involved in the removal of p27, furthering the proliferation of CECs^51^,^52^. To reveal the mechanism for CEC proliferation driven by RNase 5 as shown in Fig. 6, we investigated the alteration of positive and negative G_1_ regulators in cultured human CECs treated with RNase 5. In cultured human CECs, RNase 5 up-regulated phosphorylation of p27Kip1 and induced the expression of cyclins D1, D3, and E. These effects were reversed by blockade of PI3-k signaling using LY294002, or by inhibition of nuclear translocation of RNase 5 using neomycin (Fig. 7). The enhancement of cyclin D1 and D3 by RNase 5 (Fig. 7B,C) was less prominent than that of cyclin E (Fig. 7D). In addition, neomycin did not suppress RNase 5-induced cyclin D1 (Fig. 7B) unlike cyclin D3 and E, suggesting that activation of cyclin D1 by RNase 5 occurs through PI3-k/Akt signaling itself rather than nuclear localization of RNase 5 in CECs.

**Figure 8:**
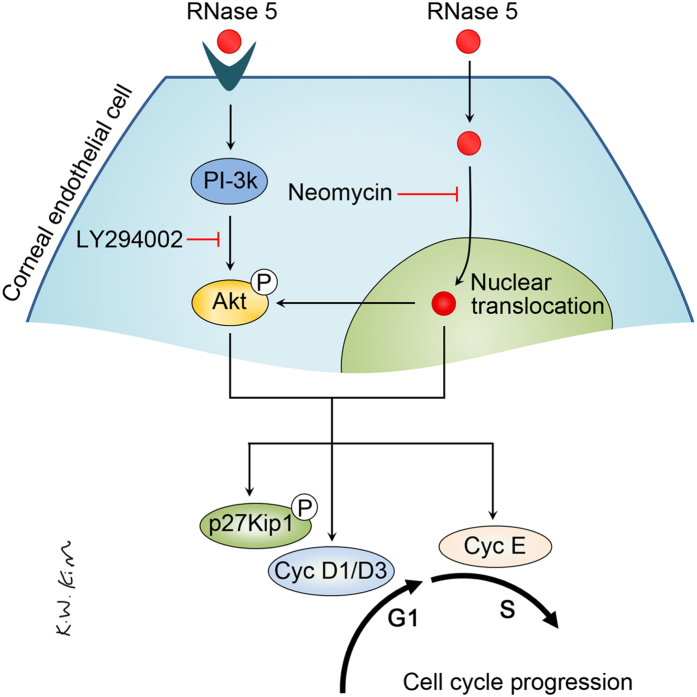
Proposed mechanism of mitotic pathways in human corneal endothelial cells induced by ribonuclease (RNase) 5-elicited PI3-kinase/Akt activation and by nuclear translocation of RNase 5.

Based on these results, we speculated that RNase 5 activates PI3-k/Akt signaling in CECs via its ability to undergo nuclear localization, ultimately leading to cell cycle progression and cellular proliferation (Fig. 8).

## Discussion

Finding a therapeutic pharmaceutical to restore the function of damaged corneal endothelia has long been a goal of ophthalmologists due to the invasiveness and risk of corneal transplantation, which is the only current surgical treatment option. However, no definite regenerative zone has been ascertained in the human corneal endothelium. Therefore, the ability to maintain CEC viability and promote proliferation is in high demand as an alternative to waiting for passive recovery by a homeostatic mechanism that may not exist in the corneal endothelia. In the present study, we identified a role of RNase 5 not previously implicated in human CECs. In brief, treatment of CECs with exogenous human RNase 5 led to strong localization of RNase 5 in the cytoplasm and nucleus in cultured human CECs. Moreover, application of RNase 5 enhanced corneal endothelial wound healing both *in vitro* and *in vivo* corneal endothelial wound models. Furthermore, we demonstrated that these actions of RNase 5 were likely due to enhanced PI3-k/Akt signaling-mediated cell cycle progression in CECs.

Unlike general wound healing mechanisms, the wound repair process observed in the human corneal endothelium is accomplished not by cell proliferation, but instead by migration of neighboring CECs^53^. Thus, the abundant cell population on the flattened monolayer of the corneal endothelium is not protected against injury or disease^54^. Moreover, the excessive cellularity of the corneal endothelium would rather induce refractive alteration or blurring that threatens the vision as seen in the retrocorneal membrane, a representative vision-threatening corneal endothelial pathologic condition. We hypothesized that the unsatisfactory proliferative capacity of human CECs may be evolutionarily linked to several necessary mechanisms for anti-proliferation to maintain corneal endothelium as one definite layer, including the low level of growth factors in aqueous humor, lack of autocrine growth factors produced by CECs, and blocked G_1_/S phase transition in CECs^2^,^11^,^55^. However, there may be some potential proliferative capacity in CECs when exposed to extraordinary conditions, such as trauma or invasive eye surgery. In support of this idea, recent reports have demonstrated the possible presence of stem cell niches in the extreme periphery of the human corneal endothelium^13^. In addition, rho-kinase inhibitors have been investigated as factors that stimulate the proliferation of human and monkey CECs^56^. In the present study, we postulated two separate mechanisms responsible for the homeostasis of the human corneal endothelium: 1) anti-proliferation of CECs in ordinary healthy conditions and 2) unlocking mitosis of CECs under wound conditions.

RNase 5 has been studied with respect to tumor growth and the pathogenesis of several cancer types. Moreover, the PI3-k/Akt pathway, which was activated by RNase 5 in this study, is known significantly involved in regulating growth, but is responsible for tumorigenesis. In this context, it is notable that treatment with RNase 5 increased the number of BrdU-incorporating CECs to a significantly greater degree in wound conditions than under confluency (Fig. 6C). Although RNase 5 significantly elevated proliferative capacity in confluent CECs, the increase in the level of BrdU incorporation was not distinct compared to controls. In addition, after RNase 5 treatment, Ki-67-expressing CECs were mainly confined to the migrating cells adjacent to the wound scrape (Supplementary Fig. S6). Together, these results suggest that the growth effect of RNase 5 on CECs follows the contact-inhibition theory, and that RNase 5 treatment is unlikely to induce tumor formation through the uninhibited and persistent proliferation of CECs, especially when the corneal endothelium is nearly healed or normal.

A clear difference following RNase 5 treatment in *ex vivo* corneal endothelial tissues as well as cultured CECs was the intracellular differential localization of RNase 5. Prior to treatment, the intracellular expression of RNase 5 was confined to the cytoplasm (Fig. 1A), while RNase 5 expression became dominant in the nucleus with accumulation in the putative nucleolus after RNase 5 treatment. This observation was consistent with reports that RNase 5 undergoes nuclear translocation and accumulates in the nucleolus, where it is involved in rRNA transcription during proliferative growth in vascular endothelial^16^, cancer^19^ and neuronal cells^31^. We postulated PI3-k/Akt signaling as one of the key pathways for corneal endothelial wound healing which, importantly, occurs via nuclear localization of RNase 5 in CECs.

*In vivo* human CECs express p27Kip1^3^,^4^, and the action of p27Kip1 differs depending on its subcellular localization^57^. P27Kip1 controls cells in the G_1_ phase by binding to cyclin D or E complexes in the nucleus, but can no longer inhibit cell proliferation as it is phosphorylated and degraded upon being exported from the nucleus into the cytoplasm. Blocking the nuclear translocation of RNase 5 inhibited the p27Kip1 phosphorylation in human CECs (Fig. 7A). This result indicated that RNase 5, in the nucleus, may affect the nuclear export of p27, thereby activating cyclin D and E in CECs. Notably, *ex vivo* expression of RNase 5 protein are predominantly in the cytoplasm of normal human corneal endothelial tissues (Fig. 1A). We suggest that resident endogenous RNase 5 in CECs exists mainly in the cytoplasm in the stable, unthreatened corneal endothelium to suppress cell cycle progression by keeping the action of negative regulators, including p27Kip1, thereby maintaining contact-inhibition.

Decompensated corneal endothelial tissues included many CECs with defective overall intracellular expression of RNase 5 protein (Fig. 1B). Based on copious cytoplasmic existence of RNase 5 in CECs from normal tissues (Fig. 1A), our results suggest that the varied degrees of deficiency of RNase 5 in CECs from decompensated tissues may have been due to consumption of pre-existing intracellular RNase 5 promoting survival or proliferation of CECs. However, the correlation between intracellular shortage of RNase 5 and limitation of proliferation of CECs should be confirmed in future *in vivo* studies.

This is the first study to demonstrate an effect of RNase 5 on the mitosis of human CECs. In addition, this is the first report to show RNase 5-induced activation of mitosis-related factors including cyclin D1, D3, and E. Although it is unclear why RNase 5 activated cyclin E much more prominently than cyclin D1 and D3, one possibility is that cyclin D and E control different events in addition to having an additive effect on shortening the G_1_ interval as previously reported^58^.

For the purpose of exploring RNase 5-elicited pathways other than PI3-k/Akt in CECs, we investigated signaling pathways known to enhance cell proliferation, including mTOR, Erk, and inhibition of rho-kinase^56^ (Supplementary Fig. S8). Unexpectedly, RNase 5 treatment did not affect the phosphorylation of Erk and P70s6k (the downstream target of mTOR) and only moderately inhibited myosin phosphatase target subunit 1 (MYPT1, the downstream target of rho-kinase). This result was inconsistent with the role of RNase 5 in human trabecular meshwork cells reported in our previous study^59^. Thus, it seems that RNase 5-elicited signals differ according to the type of ocular cell. We expect that future genome sequencing or microarray analysis with RNase 5 may aid in discovering other promising target specific for proliferation of CECs.

One limitation of this study was that we only used LY294002 to inhibit PI3-k specifically and we applied LY294002 at a fixed concentration (20 μM). Several previous reports^46^,^47^,^56^,^60^ have used LY294002 at the known optimal concentration of 20 μM^61^ to inhibit PI3-k in CECs. Furthermore, LY294002 does not inhibit other kinases at higher concentrations (50 μM) according to its product data sheet. Nevertheless, specific inhibition of PI3-k in our study should have been confirmed using other PI3-k inhibitors to reduce concerns that LY294002 may have inhibited other targets such as mTOR to trigger unexpected feedback mechanisms involving Akt activation.

In future studies, RNase 5-induced enhancement of wound healing should be reproduced in the eyes of primate mammals, because rabbit CECs are known to be capable of undergoing proliferation. However, we identified that RNase 5 is expressed in the normal human corneal endothelium and is capable of unlocking the halted mitosis of CECs via its nuclear accumulation and activation of PI3-k/Akt signaling. Thus, our results contribute to an increased understanding of a long-standing problem in the management of corneal endothelial diseases. In our opinion, co-treatment of RNase 5 in conjunction with future CEC transplantation or pre-established endothelial keratoplasty may result in higher rates of successful treatment.

In conclusion, with respect to RNase 5 treatment-elicited enhancement of wound healing in both *in vitro* and *in vivo* corneal endothelial wound models, RNase 5 may provide a mechanism to restore the injured corneal endothelium and is thus a useful target for therapeutic exploitation.

## Methods

### Materials, reagents and antibodies

Detailed information of product numbers and commercial suppliers of materials, reagents and antibodies used in this study is listed in the Supplementary Methods.

### Whole mount staining of cadaveric corneal tissue

Human corneal tissues from the control group (normal) and patient group with decompensated corneal endothelium, were obtained for research and central 8 mm round corneal tissues excised were stored at 4 °C in storage medium (Optisol-GS, Bausch & Lomb, Rochester, NY, USA). When treating with RNase 5, the corneal tissues were stored in Endothelial Growth Medium (EGM)-2MV BulletKit^TM^ (Lonza, Walkersville, MD, USA) medium with human RNase 5 (5 μg/mL) for 6 hours at 37 °C in an incubator. Next, cornea tissues were fixed in 4% paraformaldehyde at 4 °C overnight and washed 3 times with phosphate-buffered saline (PBS) at room temperature (RT). Corneal endothelium was then permeabilized by incubation for 20 minutes at RT with PBS supplemented with 0.5% Triton X-100 and washed by rocking 3 times in PBS. Subsequent steps are described in the Supplementary Methods.

The expression of RNase 5 in CECs of human corneal endothelial tissues was determined based on standard photos of CECs expressing RNase 5 (small white rectangle in Fig. 1A) or defective intracellular RNase 5 (small white rectangles in Fig. 1B).

### Animals

New Zealand White rabbits (total 24 eyes) weighing 2.0 to 3.0 kg and raised at the Clinic Research Center, Chung-Ang University, College of Medicine, were used and in this study. Animals were assigned to one of three groups: control group, RNase 5 treatment group, and a LY294002 + RNase 5 treatment group.

### Preparation of cryo-damage of corneal endothelium in rabbit model

A combination of tiletimine and zolazepam-mixed agent (Zoletil^TM^, Virbac, Fort Worth, TX, USA) and xylazine (Rompun^TM^, Bayer, Leverkusen, Germany) was injected intramuscularly for anesthesia (12.5 mg/kg). The corneal endothelium of rabbits was cryo-damaged according to the established protocol by Okumura *et al*.^62^. In brief, a stainless steel probe with a diameter of 7 mm was immersed in liquid nitrogen tank for 3 minutes to achieve a stabilized temperature of approximately −196 °C. Thereafter, corneal endothelium was damaged by transcorneal freezing by gently touching the central corneal surface with the probe for 15 seconds as noted in Fig. 3C. The ocular surface was then irrigated using 10 mL of normal saline. Topical levofloxacin eye drops (Cravit^TM^, Santen, Osaka, Japan) were applied 4 times a day for 3 days to prevent infection.

### Instillation of eye drops in rabbit model

RNase 5 (200 μg/mL) eye drops and PBS eye drops as a control were applied 6 times (50 μL in each application) a day for 48 hours, and then 4 times a day until 192 hours after injury. RNase 5 was diluted in PBS.

### Analysis of corneal edema and central corneal thickness in rabbit model

Corneal edema was evaluated and central corneal thickness was measured at 48, 72, 144 and 192 hours after cryo-injury in the rabbit model. Corneal edema was evaluated under slit-lamp biomicroscopic examination. The severity of corneal edema was quantified from 0 to +4.0 according to the well-known grading system of corneal opacity (0: completely clear cornea; +0.5: faint haze, only detectable by careful oblique illumination; +1.0: minimal haze, seen with difficulty with direct illumination; +2.0: mild haze, easily visible with direct focal slit illumination; +3.0: moderately dense haze that partially obscured iris details; +4.0: severe dense haze that obscured completely iris details) established by Fantes *et al*.^63^. Central corneal thickness was measured using an ultrasound corneal pachymeter (POCKET-II, Quantel medical, Clemont-Ferrand, France; Measureable up to maximal 1,000 μm). The average of three repeated measurements was recorded for analysis.

### Analysis of corneal endothelial wound area in rabbit model

Rabbit eyes were enucleated at 192 hours and corneal button tissues were excised from the eyeball. The excised corneal tissues were then immersed in 1% Alizarin red S solution (Lab Chem, Pittsburgh, PA, USA) for 2 minutes and washed with normal saline. Next, flat-mounts were placed on glass slides with the endothelial layer facing up and photo-documented under inverted optical microscopic examination (IX71, Olympus, Tokyo, Japan). The corneal endothelial wound area was measured using Image J software ver. 1.46 (National Institutes of Health (NIH); http://rsbweb.nih.gov/ij/).

### Culture of human CECs

Human corneal tissues for corneal transplantation without previous ocular diseases were obtained and the peripheral corneal tissues after excision of central 8 mm round cornea were used for the culture of human CECs. Corneal tissues were washed 3 times in a 5× antibiotics (penicillin/streptomycin) solution followed by 6 times in PBS. Cells on the corneal endothelial layer were isolated using a peel-and-digest approach. Subsequent steps are described in the Supplementary Methods.

### Cell viability (MTT) assay

Human CECs were cultured in 96-well culture plates for 16 hours. Cell viability was assessed using the MTT assay method. Briefly, 3-(4,5-dimethylthiazol-2-yl)-2,5-diphenyltetrazolium bromide (MTT) was dissolved in PBS at a concentration of 5 mg/mL. MTT was then added to each well (10 μL per 100 μL medium) and plates were incubated at 37 °C for 2  hours in the absence of light. The medium was replaced with 100 μL dimethyl sulfoxide (DMSO), and the absorbance for each well was measured at 570 nm using a Spectramax^TM^ 340PC384 microplate photometer (Molecular Devices, Sunnyvale, CA, USA).

### Immunocytochemistry of RNase 5 and Ki-67

Human CECs were seeded at a density of 1.6 × 10^4^ cells/cm^2^ on FNC-coated 12 mm coverslips (Deckglasser, Mülheim, Germany). CECs were incubated in the presence or absence of human RNase 5 treatment (5 μg/mL) 24 hours after seeding. For RNase 5 treatment, cells were treated for 1, 3, 6 (immunocytochemistry of RNase 5), or 24 hours (immunocytochemistry of Ki-67) as indicated. Scraping using a 200 μL pipette (yellow tip) was done in some of slides to detect Ki-67-positive CECs before addition of RNase 5 to analyze the effect of RNase 5 as an *in vitro* subconfluent wound model. Subsequent steps are described in the Supplementary Methods.

### Immunocytochemistry of Na^+^/K^+^ATPase and ZO-1

Human CECs were seeded at a density of 2.0 × 10^5^ cells/cm^2^ on FNC-coated 8-well Lab-Tek II chamber glass slides (Nalge Nunc Inc., Naperville, IL, USA). CECs cultured for 2 days were washed twice with PBS and fixed with 4% paraformaldehyde for 20 minutes. Subsequent steps are described in the Supplementary Methods.

### Wound healing assay of cultured CECs

Human CECs were grown in 6-well plates to create 80 to 90% confluent monolayer. To create scratch wounds, a straight line in the cell monolayer was made by scraping with 200 μL pipette (yellow tip) in a straight line. Next, for RNase 5 treatments, CECs were treated with RNase 5 (5 μg/mL) until 36 hours. When treating with inhibitors, CECs were pre-incubated with LY294002 (20 μM) or neomycin (1 mM) for 1 hour before and during exposure to RNase 5 (5 μg/mL for 24 hours). Images of migrated cells were documented by inverted optical microscopic examination (IX71, Olympus, Tokyo, Japan) and wound areas were analyzed using Image J software. The extent of wound healing was determined by the areas of migration into the denuded area and presented as a wound healing index (%): (initial wound area-remaining wound area)/initial wound area. For the wound healing assay using mitomycin C, CECs were treated with 5 μg/mL mitomycin C for 2 hours prior to wounding in order to eliminate the influence of cell proliferation.

### BrdU cell proliferation assay

Cell proliferation was measured using the BrdU Cell Proliferation Kit (Millipore, Billerica, MA, USA) according to the manufacturer’s instructions. Human CECs were seeded at a density of 7,500 cells/well in FNC-coated 8-well Lab-Tek II chamber glass slides (Nunc, Naperville, IL, USA). To establish the scraping wound model, cells were scratched with a 200 μL pipette in the middle of wells 24 hours after seeding. For inhibitor treatments, human CECs were pre-incubated with either LY294002 (20 μM) or neomycin (1 mM) for 1 hour before and during exposure to RNase 5 (5 μg/mL for 24 hours). Subsequent steps are described in the Supplementary Methods.

### Cell growth analysis

CECs were seeded at a concentration of 1 × 10^5^ cells/mL in a 35 mm plate (SPL life sciences, Pocheon, Gyeonggi-do, Republic of Korea) and treated with or without RNase 5 (5 ug/mL). Following 24, 48, 72, 96 and 120 hours (day 5), CECs were harvested, diluted by trypan blue working solution and counted using hemocytometer.

### Western blot

When verifying the time-dependent effect of RNase 5 on Akt expression, cultured human CECs were treated with RNase 5 (5 μg/mL) for various times (5 minutes to 3 hours). When CECs were treated with inhibitors, cells were pre-incubated with either LY294002 (20 μM) or neomycin (1 mM) for 1 hour before and during exposure to RNase 5 (5 μg/mL for 24 hours). The protocol for Western blot analysis performed in this study is described in the Supplementary Methods.

### Statistics

Two-tailed Student’s *t*-test and analysis of variance (ANOVA) followed by *post-hoc* analysis were used for the statistical analysis between two groups and among three or more groups, respectively. *p* values < 0.05 were considered significant.

### Study approval

All experiments other than animal studies were approved by the Institutional Review Boards at the Chung-Ang University Hospital, and the methods were carried out in accordance with the approved guidelines. Informed consent was obtained for the use of human cadaveric ocular tissues in accordance with the Declaration of Helsinki. Animal procedures in this study were performed in accordance with ARVO Statement for the Use of Animals in Ophthalmic and Vision Research, and were approved by the Chung-Ang University Ethics Committee for Animal Experiment.

## Additional Information

**How to cite this article**: Kim, K. W. *et al*. Ribonuclease 5 facilitates corneal endothelial wound healing via activation of PI3-kinase/Akt pathway. *Sci. Rep.* **6**, 31162; doi: 10.1038/srep31162 (2016).

## Supplementary Material

Supplementary Information
