# Supplementary material for: Ribonuclease 5 facilitates corneal endothelial wound healing via activation of PI3-kinase/Akt pathway
Source: Sci Rep. 2016 Aug 16;6:31162. doi: 10.1038/srep31162 (PMC4985649; doi:10.1038/srep31162)
Supplement: Supplementary Information [file srep31162-s1.pdf]

Ribonuclease 5 facilitates corneal endothelial wound healing via activation of PI3-kinase/Akt pathway

Kyoung Woo Kim[1, 2], Soo Hyun Park[1], Soo Jin Lee[1], Jae Chan Kim[1*]

[1]Department of Ophthalmology, College of Medicine, Chung-Ang University Hospital, Seoul, Korea

[2]Graduate School of Chung-Ang University, College of Medicine, Seoul, Korea

***Corresponding author: Jae Chan Kim**

Department of Ophthalmology, Chung-Ang University Hospital, 224-1, Heukseok-dong, Dongjak-gu, Seoul 156-755, Korea

Tel.: 82-2-6299-1689, Fax: 82-2-825-1666

E-mail: jck50ey@daum.net

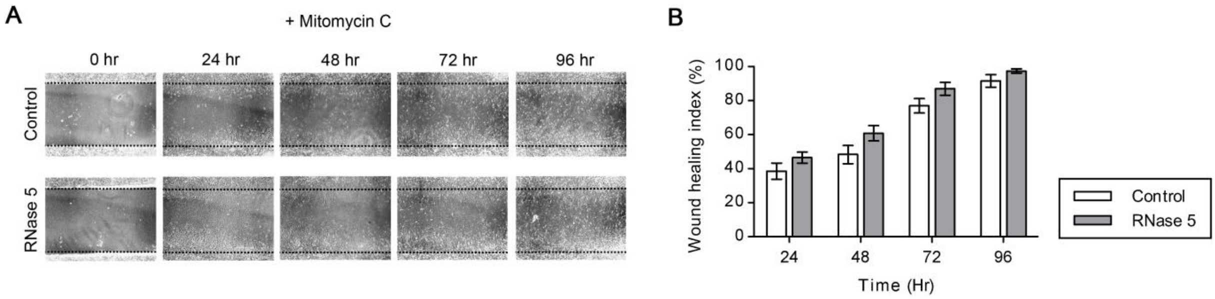

Supplementary Fig. S1. The *in vitro* wound healing of cultured corneal endothelial cells (CECs) pretreated with mitomycin C in the ribonuclease (RNase) 5 and in the control group.

(**A**) The remaining wound area inside the initial wound (between black dotted lines) was shown in the RNase 5 (5 μg/mL) group and in the control. (**B**) Although the wound healing index with RNase 5 showed higher tendency compared to without RNase 5, there was no significant difference between two groups over 96 hours. *n* = 4 independent experiments.

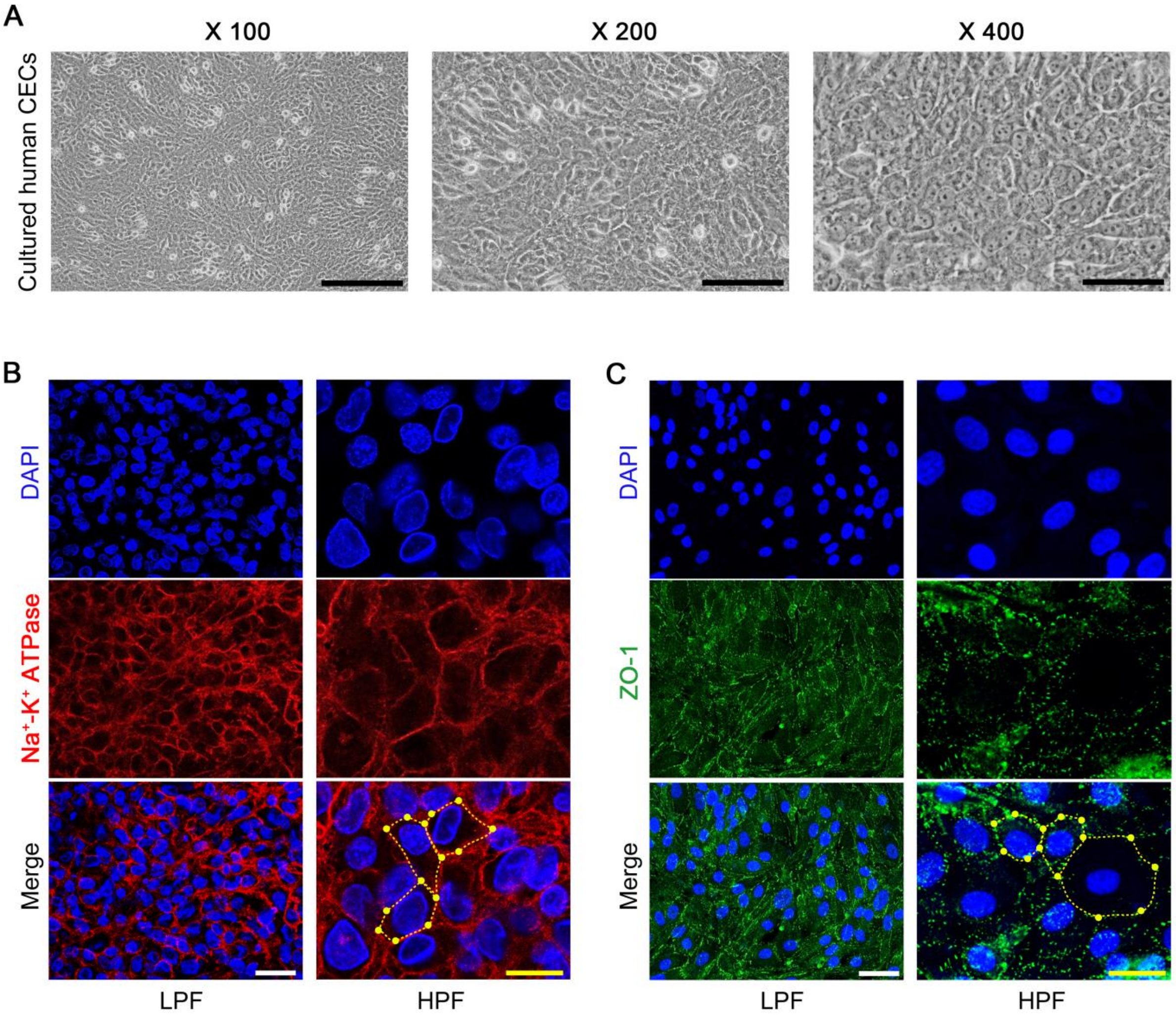

Supplementary Fig. S2. Representative images of cultured human corneal endothelial cells (CECs) and images of Na^{+}-K^{+} ATPase and zonula occludens (ZO)-1 expressions in CECs.

(**A**) Images of different magnifications of human cultured CECs used in the experiments in the present study. Confluent CECs formed junctions well each other. Scale bars: 200 μm (x 100), 100 μm (x 200), 50 μm (x 400). (**B, C**) Images of low-power field (LPF) and high-power field (HPF) views of cultured human CECs expressing Na^{+}-K^{+} ATPase (**B**) and ZO-1 (**C**) defined by immunofluorescence assay. CECs expressed Na^{+}-K^{+} ATPase and ZO-1 along hexagon-shaped cell membrane. Representative CECs expressing Na^{+}-K^{+} ATPase and ZO-1

on their membranes were shown in HPF images (boundaries of yellow dotted lines). Scale bars: 50 μm (white) and 20 μm (yellow).

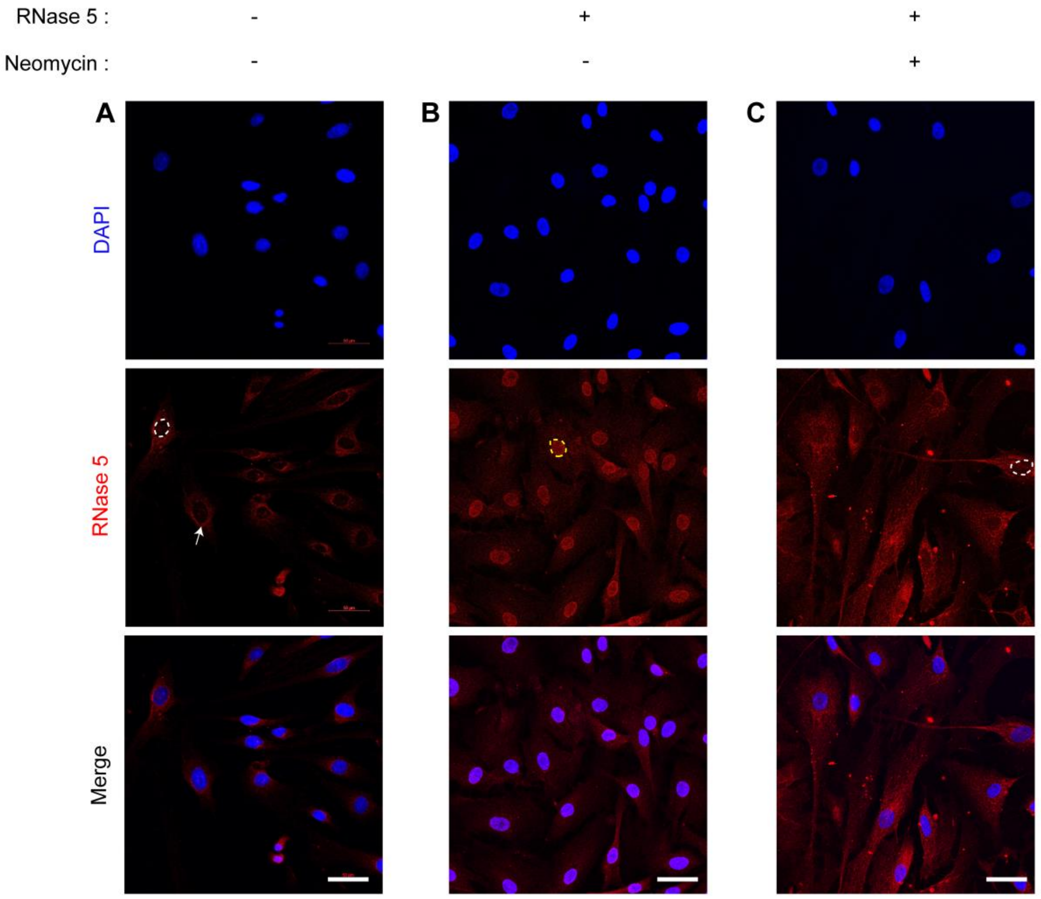

Supplementary Fig. S3. Immunofluorescence of ribonuclease (RNase) 5 in cultured human corneal endothelial cells (CECs) treated with or without RNase 5, and in the absence or presence of neomycin.

(**A**) Before the treatment of RNase 5 or neomycin (as a control), CECs revealed subtle expression of intracellular RNase 5 at the perinuclear cytoplasmic area (arrow) and lacked nuclear expression of RNase 5 (white dotted circle) same as described in Fig. 4B. (**B**) Treatment of CECs with RNase 5 (5 μg/mL, 6 hours) induced prominent nuclear accumulation of RNase 5 fluorescence (yellow dotted circle). (**C**) Co-treatment of neomycin (1 mM) suppressed RNase 5-induced nuclear expression of RNase 5. Reversed intracellular RNase 5 fluorescence in a CEC was representatively shown in the white dotted circle. Scale bars: 50 μm.

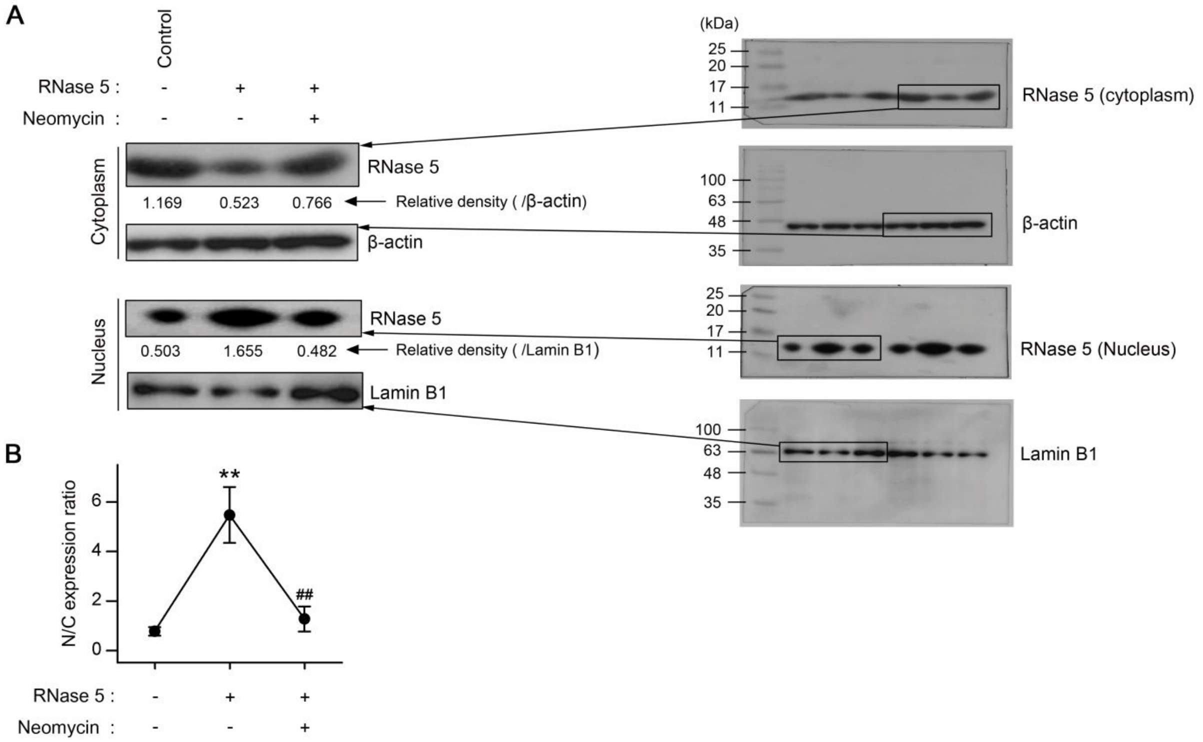

Supplementary Fig. S4. Immunoblotting of nuclear and cytoplasmic fractions of ribonuclease (RNase) 5 in cultured human corneal endothelial cells (CECs).

(**A**) Representative immunoblotting and quantitative analysis of band density of cytoplasmic and nuclear RNase 5 in cultured human CECs treated with or without RNase 5 (5 µg/mL, 6 hours), and in the presence or absence of neomycin (1 mM) co-treatment. Co-treatment with neomycin (1 mM) in CECs inhibited expression of nuclear fraction of RNase 5, on the contrary increased that of cytoplasmic fraction after treatment of CECs with RNase 5. (**B**) The ratio of nuclear amount of RNase 5 to that of cytoplasmic fraction (N/C expression ratio) according to Western blots was described. Co-treatment of CECs with neomycin decreased the ratio of N/C expression of RNase 5. **p = 0.004, vs. control. $^{\#\#}p$ = 0.008, RNase 5 treatment with vs. without neomycin. n = 4 independent experiments. β-actin and Lamin B1 were used as the loading controls in cytoplasmic and nuclear expressions, respectively.

Cropped gels are accompanied by full-length gels. Statistical analysis was performed with ANOVA followed by Bonferroni's *post-hoc* analysis. Values represent the mean $\pm$ s.e.m.

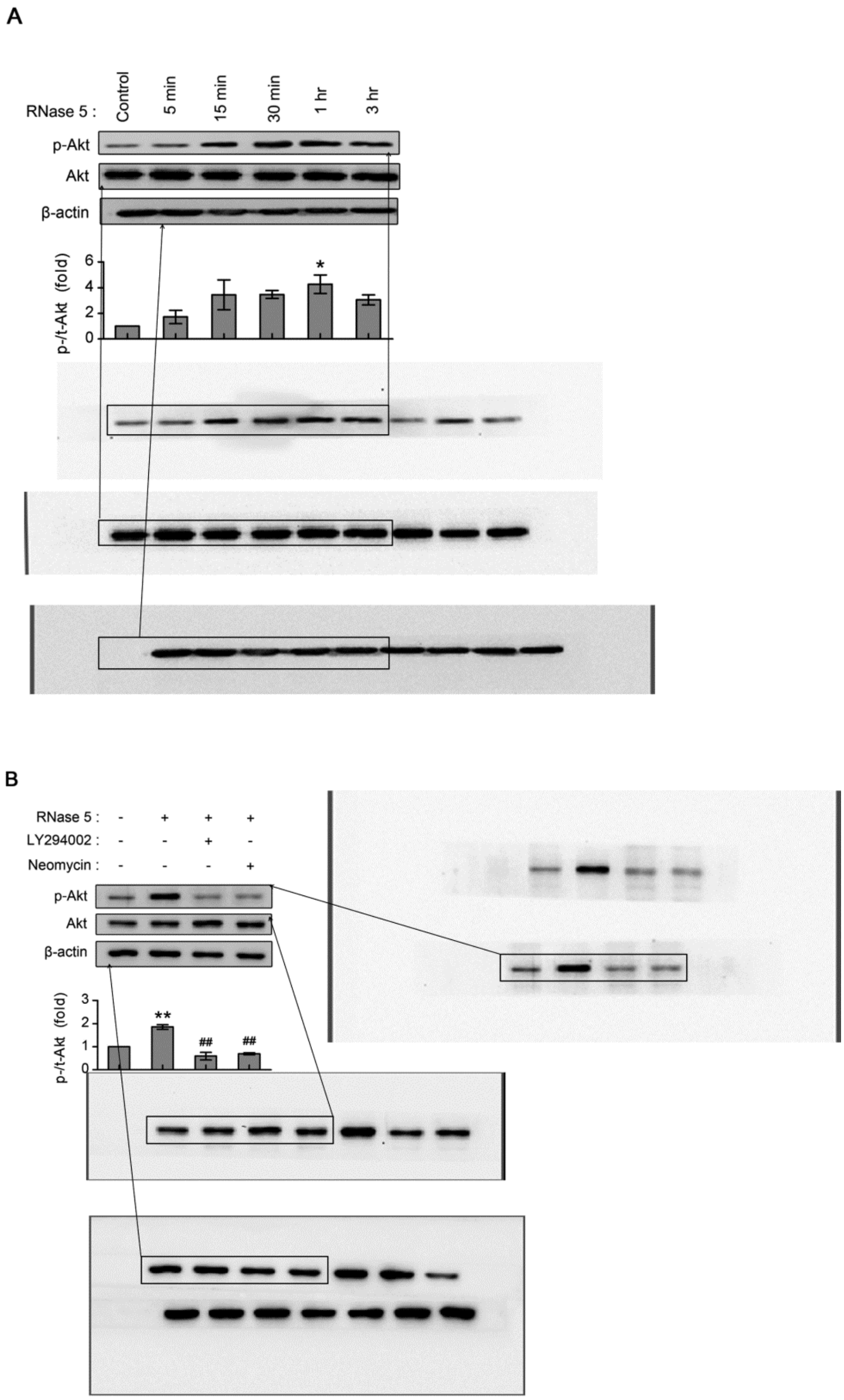

Supplementary Fig. S5. Full length-gels of cropped gels in figure 5A and B.

Full-length gels of the representative gels in figure 5A and 5B are shown in panels (**A**) and (**B**), respectively.

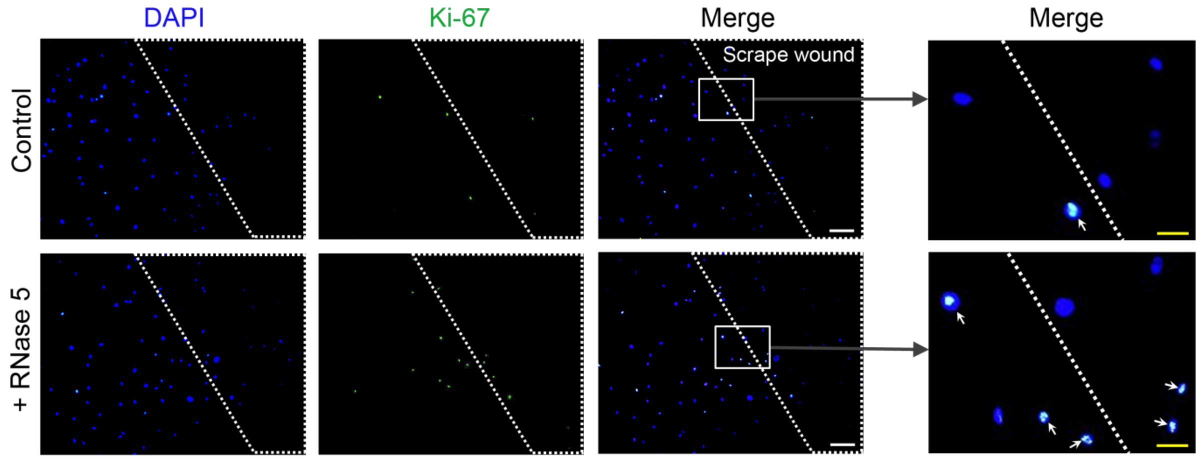

Supplementary Fig. S6. Representative images of Ki-67 staining of cultured human corneal endothelial cells (CECs) with or without ribonuclease (RNase) 5 treatment after scape wounding.

Ki-67 expression was concentrated in CECs adjacent to the scraped margin in the RNase 5 group (5 μg/mL for 24 hours). In magnified images of small white rectangles, the Ki-67^{+} cells next to the scrape wound border (dotted line) with or without RNase 5 treatment are indicated (arrows). Scale bars: 200 μm (white), and 50 μm (yellow). *n* = 3 independent experiments.

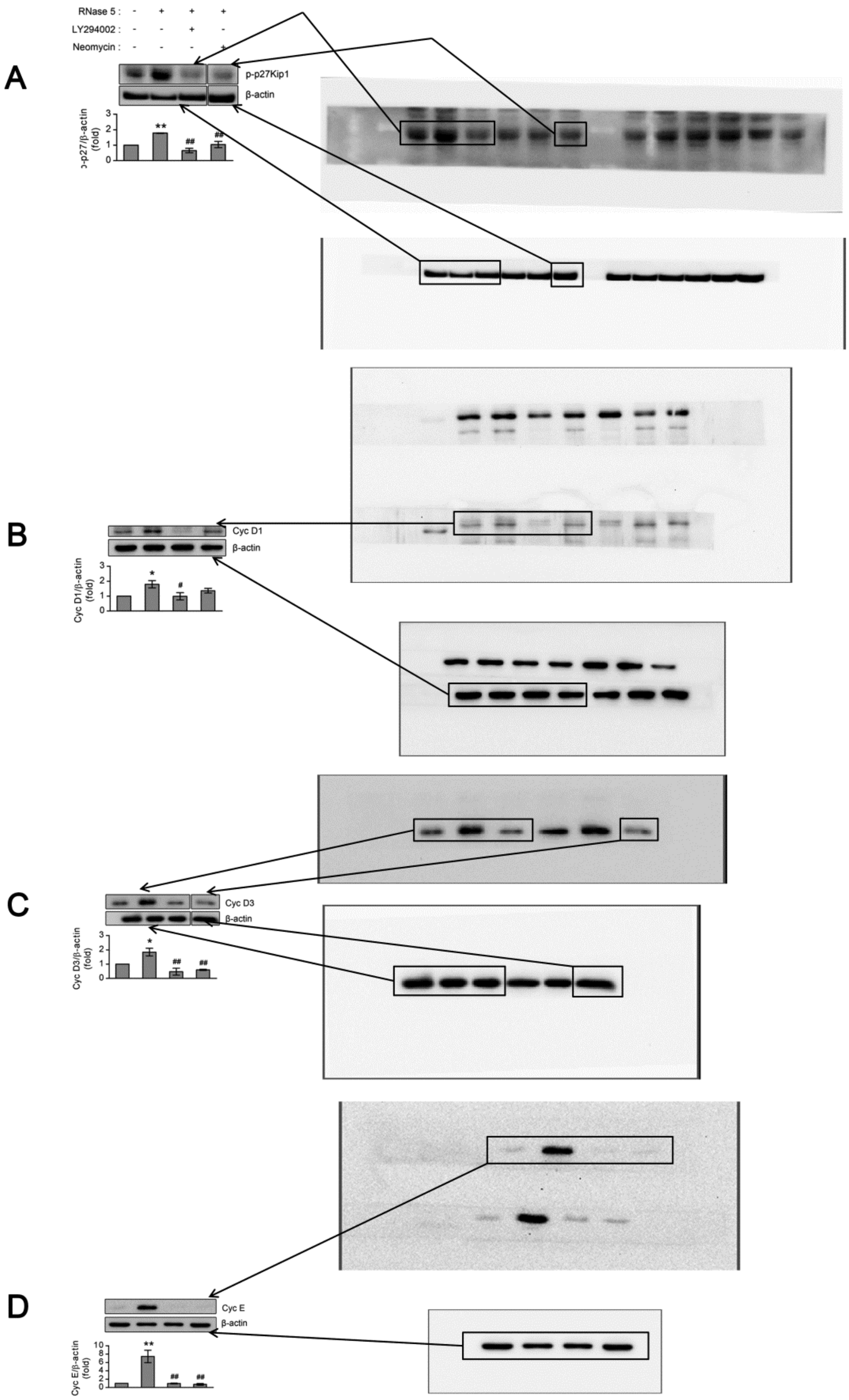

Supplementary Fig. S7. Full length-gels of cropped gels in figure 7.

Full-length gels of the representative gels in figure 7A to 7D are shown in panels (**A**), (**B**), (**C**), and (**D**), respectively.

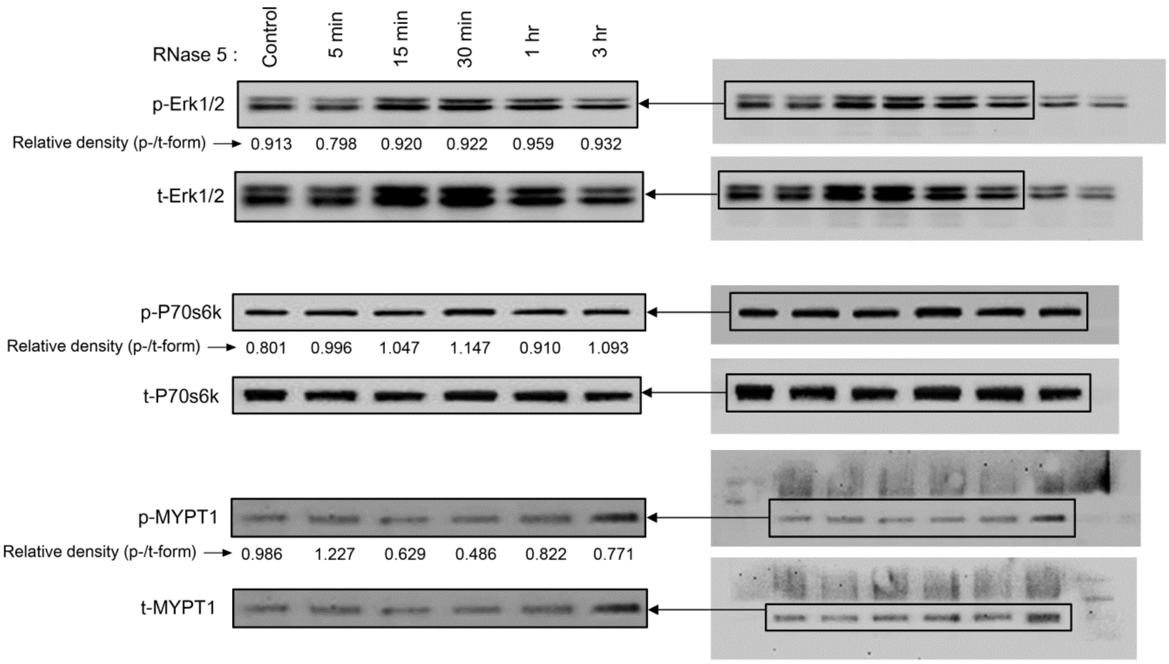

Supplementary Fig. S8. Immunoblotting of Erk1/2, P70s6k, and myosin phosphatase target subunit 1 (MYPT1) in cultured human corneal endothelial cells (CECs) with or without ribonuclease (RNase) 5 treatment.

The amount of phosphorylated Erk1/2 (p-Erk1/2) and P70s6k (p-P70s6k) relative to that of total Erk1/2 (t-Erk1/2) and P70s6k (t-P60s6k) was not increased by treatment with RNase 5 (5 μg/mL) for 3 hours in CECs. MYPT1 phosphorylation was very slightly inhibited with RNase 5 (5 μg/mL) at 15 and 30 minutes. Cropped gels are accompanied by full-length gels.

Supplementary Methods

Materials, reagents and antibodies.

Throughout this study, the following materials, reagents and antibodies (Abs) were used: human recombinant RNase 5 protein (265-AN/CF, R&D Systems, Minneapolis, MN, USA), LY294002 (BML-ST420-0025, Enzo Life Sciences, Farmingdale, NY, USA), neomycin (3573, Santa Cruz, CA, USA), Abs; mouse monoclonal Abs (mAbs) anti-RNase 5 (14017.7, Abcam, Cambridge, MA, USA), anti-Na^{+}/K^{+} ATPase (464.6, Abcam), anti-cyclin E (4129, Cell Signaling Technology, Danvers, MA, USA), anti-β-actin (5441, Sigma-Aldrich, St. Louis, MO, USA), rabbit mAbs anti-p-p27Kip1 (phosphor T187, 75908, Abcam), anti-cyclin D1 (2978, Cell Signaling Technology), anti-cyclin D3 (52598, Abcam), anti-Erk1/2 (4695, Cell Signaling Technology), anti-p-Erk1/2 (4370, Cell Signaling Technology), anti-P70s6k (2708, Cell Signaling Technology), anti-p-P70s6k (9234, Cell Signaling Technology), rabbit polyclonal Abs (pAbs) anti-Akt (9272, Cell Signaling Technology), anti-p-Akt (9271, Cell Signaling Technology), anti-MYPT1 (2634, Cell Signaling Technology), anti-p-MYPT1 (5163, Cell Signaling Technology), anti-Ki-67 (15580, Abcam), anti-Lamin B1 (16048, Abcam), anti-zonula occludens (ZO)-1 (40-2200, Invitrogen, Waltham, MA, USA), and secondary Abs conjugated with fluorescein isothiocyanate (FITC) (AffiniPure goat anti-rabbit IgG, 111-095-144 & anti-mouse IgG 115-095-166, Jackson Immuno-Research, West Grove, PA, USA) and Cy3 (AffiniPure goat anti-mouse IgG, 115-165-166, Jackson Immuno-Research).

Whole mount staining of cadaveric corneal tissue.

Non-specific binding was blocked using 2% bovine serum albumin (BSA) in PBS (pH 7.4) for 30 minutes at RT. Corneal tissues were transferred to 0.2 mL anti-RNase 5 Ab

solution (1:50 in PBS with 2% BSA) for 3 hours at 37 ℃ incubator and rinsed 3 times in PBS, and then incubated with 0.2 mL secondary Ab (conjugated with FITC, 1:100 in PBS and 2% BSA) for 1 hour at 37 ℃. Next, corneas were washed 3 times in PBS and flat-mounted with a glass coverslip. Flat-mounts were placed on glass slides with the endothelial layer facing up and mounted in Fluoroshield™ mounting medium with DAPI (Sigma-Aldrich).

Culture of human corneal endothelial cells (CECs).

Using sterile surgical forceps, the sheet of descemet's membrane with intact endothelium was carefully peeled, incubated in 0.25% trypsin/EDTA at 37 ℃ for 10 minutes, and then pipetted for 2 to 3 minutes to dislodge CECs from the descemet's membrane. Detached CEC-clusters were rinsed once in EGM-2MV BulletKit™ and further dissociated to obtain smaller cell-clumps. The cell clumps were washed and collected after centrifugation at 1,200 rpm for 5 minutes and plated on FNC-coated tissue culture dishes for attachment. Isolated cells were cultured in EGM-2MV BulletKit™. When the CECs reached 80 to 90% confluency, they were passaged using trypsin/EDTA and sub-cultured at a seeding density of 10,000 cells/cm^2. Experiments were performed using cells at the third passage. All incubation and cultures of human CECs were carried out in a humidified incubator at 37 ℃ with 5% CO_2 and fresh medium was replenished every two days.

BrdU proliferation assay and Western blot analysis for cell-cycle related factors (p-p27Kip1 and cyclins D1, D3, and E) were conducted using cultured human CECs that had been subjected to growth factor/serum-deprived starvation in endothelial basal medium (EBM)™-2 medium (Lonza) supplemented with only 1% FBS for 4 hours.

Immunocytochemistry of RNase 5 and Ki-67.

Human CECs were fixed with 4 % paraformaldehyde for 20 minutes and permeabilized with 0.5 % Tween-20 in PBS (pH 7.4) for 30 minutes. To prevent non-specific binding, the slides were incubated with a 5% normal goat serum blocking agent (Vector Laboratories Inc., Burlingame, CA, USA) in PBS (pH 7.4) for 1 hour. Slides were incubated with primary Abs against RNase 5 (1:250 in PBS containing 1% normal goat serum and 0.1% Triton X-100) or Ki-67 (1:250 in PBS containing 1% normal goat serum and 0.1% Triton X-100) overnight at 4 °C. Next, the slides were incubated with secondary Ab conjugated with FITC (1:200 in PBS with 5% normal goat serum) for 2 hours at RT. Flat-mounts were placed on glass slides with the endothelial layer facing up and mounted in Fluoroshield™ mounting medium with DAPI to visualize nuclei and inhibit fading of the immunolabeling. Immunofluorescence images under the inverted optical microscopic were photo-documented. In the case of RNase 5 images, the data were processed by converting the images into a gray scale and thresholding the image at a lightness of 30. Counting of K-67-positive CECs was performed using Image J software.

Immunocytochemistry of Na^+/K^+ ATPase and ZO-1

Non-specific binding was blocked using 10% normal goat blocking solution in PBS (pH 7.4) for 1 hour at RT, followed by overnight incubation with anti-Na^+/K^+ ATPase Ab (1:100 in PBS containing 1% normal goat serum, 0.1% Triton X-100) or anti-ZO-1 (1:100). Next, the slides were incubated with secondary Ab conjugated with Alexa Fluor 568 (Invitrogen, 1:300 in PBS with 5% normal goat serum) for 2 hours at RT. Flat-mounts were placed on glass slides with the endothelial layer facing up and mounted in Fluoroshield™ mounting medium with DAPI. The slides were then examined using a confocal microscope (Zeiss LSM 700, Carl Zeiss, Jena, Germany).

BrdU cell proliferation assay

BrdU was added 8 hours before the end of the RNase 5 treatment period. Human CECs were then fixed and incubated with mouse anti-BrdU Ab followed by incubation with a peroxidase-conjugated anti-mouse Ab. Color was developed by the addition of 3,3′,5,5′-tetramethylbenzidine peroxidase substrate. After incubation for 30 minutes, the reaction was stopped with stop solution. BrdU incorporation was measured at 450 nm using a Spectramax™ 340PC384 microplate photometer.

Western blotting

Cells were washed twice with PBS and lysed with PRO-PREP™ (iNtRON Biotechnology Inc., Gyeonggi-do, Republic of Korea). Cell lysates were separated on sodiumdodecyl sulfate (SDS)-polyacrylamide gels and transferred to polyvinylidene fluoride (PVDF) membrane (Millipore, Bedford, MA, USA). Nonspecific Ab binding was blocked with 3% BSA in TBS-T (50 mM Tris-HCl pH 7.5, 150 mM NaCl and 0.1% Tween-20) for 1 hour at RT. Primary Abs against RNase 5, Akt, p-Akt, p-p27, cyclin D1, cyclin D3, cyclin E, Lamin B1 and β-actin diluted in TBS-T containing 5% BSA (1:1,000 except RNase 5, which was 1:250) were applied to the membrane, and incubated overnight at 4 °C. The membranes were incubated with the appropriate secondary Abs diluted in TBS-T containing 5% skim milk (1:3,000) for 1 hour at RT. The binding of specific Abs was visualized using an enhanced chemiluminescence Western Blotting detection kit (Pierce Biotechnology, Inc., Rockford, IL, USA). For Western blotting of nuclear and cytoplasmic RNase 5, nuclear and cytoplasmic proteins were isolated using the EpiXtract™ Nuclear Protein Isolation Kit (Enzo Life Sciences) according to the manufacturer's instructions. The value of each band was

normalized to that of the corresponding β-actin signal. Quantification of immunobands was performed using Image J software.
